# Recognition competes with hydration in anion-triggered monolayer formation of cyanostar supra-amphiphiles at aqueous interfaces†

**DOI:** 10.1039/d2sc00986b

**Published:** 2022-03-15

**Authors:** Liwei Yan, Ankur Saha, Wei Zhao, Jennifer F. Neal, Yusheng Chen, Amar H. Flood, Heather C. Allen

**Affiliations:** Department of Chemistry and Biochemistry, The Ohio State University Columbus Ohio 43210 USA allen@chemistry.ohio-state.edu +1-614-292-1685 +1-614-292-4707; Department of Chemistry, Indiana University Bloomington Indiana 47405 USA aflood@indiana.edu +1-812-855-8300 +1-812-856-3642

## Abstract

The triggered self-assembly of surfactants into organized layers at aqueous interfaces is important for creating adaptive nanosystems and understanding selective ion extraction. While these transformations require molecular recognition, the underlying driving forces are modified by the local environment in ways that are not well understood. Herein, we investigate the role of ion binding and ion hydration using cyanosurf, which is composed of the cyanostar macrocycle, and its binding to anions that are either size-matched or mis-matched and either weakly or highly hydrated. We utilize the supra-amphiphile concept where anion binding converts cyanosurf into a charged and amphiphilic complex triggering its self-organization into monolayers at the air–water interface. Initially, cyanosurf forms aggregates at the surface of a pure water solution. When the weakly hydrated and size-matched hexafluorophosphate (PF_6_^−^) and perchlorate (ClO_4_^−^) anions are added, the macrocycles form distinct monolayer architectures. Surface-pressure isotherms reveal significant reorganization of the surface-active molecules upon anion binding while infrared reflection absorption spectroscopy show the ion-bound complexes are well ordered at the interface. Vibrational sum frequency generation spectroscopy shows the water molecules in the interfacial region are highly ordered in response to the charged monolayer of cyanosurf complexes. Consistent with the importance of recognition, we find the smaller mis-matched chloride does not trigger the transformation. However, the size-matched phosphate (H_2_PO_4_^−^) also does not trigger monolayer formation indicating hydration inhibits its interfacial binding. These studies reveal how anion-selective recognition and hydration both control the binding and thus the switching of a responsive molecular interface.

## Abstract 2

The selective binding of anions transform macrocycles into amphiphiles that form monolayers on the surface of water depending upon the relative roles of anion hydration and anion recognition.

## Graphical abstract

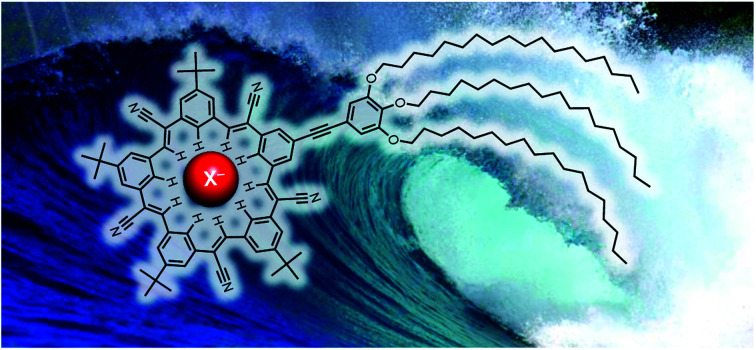

## Introduction

**Fig. 1:**
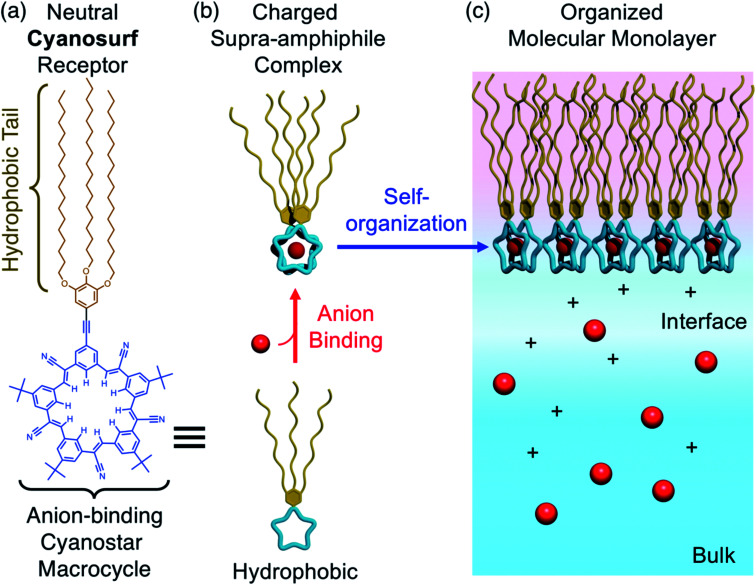
(a) Molecular design and structure of the anion-binding cyanosurf macrocycle. (b) Anion-driven transformation of cyanosurf into a supra-amphiphile by formation of a 2 : 1 complex with surfactant properties. (c) Molecular recognition by cyanosurf at the interface allows the charged supra-amphiphile to spontaneously self-organize into a monomolecular film (monolayer) at the air–water interface.

Supra-amphiphiles^1–3^ are an emerging class of surfactants that become surface active and self-organize into monolayers, bilayers, micelles, and liposomes upon the pre-programmed binding of another molecule or ion. Their responsive organization^4^ is believed to impact diverse areas ranging from the creation of adaptive materials for aqueous nanosystems, like artificial light-harvesting systems,^5^ drug delivery,^6,7^ and vesicle nanoenzymes,^8^ through to the third-phase behaviors of separation systems^9^ occurring in the liquid–liquid extraction of ions^10^ used in the nuclear fuel cycle^11^ and in the capture of critical elements.^12,13^ For example, inverse micelles form when amphiphiles extract anionic complexes of lanthanide ions^14^ from nitrate-rich aqueous solutions to organic solutions. A recent example^15^ showed the triggered transformation of a monolayer into a bilayer upon ion binding at the air–water interface. Some of these triggered phase changes do not follow expected trends and are instead ion-specific^16^ suggesting that we have an incomplete understanding of how the underlying ion-binding events are modified by the aqueous interface.^17–23^ While there is a kinship between binding-triggered extraction surfactants and supra-amphiphiles, the extraction systems do not typically use complementary receptors. Similarly, supra-amphiphiles are not routinely studied using well-defined monolayers. Thus, we see an opportunity to better understand the underlying driving forces by combining pre-programmed ion-binding sites with formation of Langmuir monolayers.^24^ Herein, we use the well-known recognition chemistry of cyanostar macrocycles^25^ to examine how the supra-amphiphile cyanosurf (Fig. 1a) self-organizes into monolayers upon the binding of specific anions (Fig. 1b and c). We leverage the selective binding of larger anions like perchlorate (ClO_4_^−^), which serves as an analog of the radioactive pertechnetate (TcO_4_^−^),^26^ and compare it to phosphate (H_2_PO_4_^−^) to set up a competition between anion recognition and anion hydration.

The self-organization of supra-amphiphiles has been programmed to respond to various recognition chemistries,^27–29^*e.g.*, host–guest binding,^30^ electrostatics,^31^ and metal cation binding.^32^ By contrast, the use of anion binding is relatively rare despite the importance of anionic species in extraction studies^12^ and in the capture of other environmentally relevant anions, *e.g.*, phosphate^33^ and nitrate (NO_3_^−^)^34^ as fertilizer, and bicarbonate (HCO_3_^−^)^35,36^ as carbon dioxide. The few examples of anion-triggered supra-amphiphiles leverage macrocycle-ion binding events. Anion–π interactions used by Wang^37^ show generation of vesicles upon binding anionic surfactants, *e.g.*, sodium dodecylsulfate. A second set of examples by Sessler use ion-pair binding. In one case, iron difluoride (FeF_2_) drives formation of micelles in aqueous solution.^31^ Another uses receptor-modified diblock copolymers that form inverse micelles under liquid–liquid extraction conditions upon binding ion pairs of alkali cations and halides, like cesium fluoride (CsF).^38^ Thus, the reliable recognition chemistry of macrocycles allow the chemical specificity of the binding event underpinning the supra-amphiphile effect^39^ to be well defined.

To complement the role of the receptor in understanding and controlling triggered phase changes, the study of monolayers at the air–water interface also serves as a well-defined model of more complex soft-matter systems.^40^ For instance, monolayers often produce greater degrees of local ordering and more well-defined interfacial regions than vesicles and micelles^41^ or the inverse micelles in the third phase.^14–16^ The analysis of monolayers also benefits from a set of complementary techniques that can reveal details of the resulting interfacial phase behavior and structures. Thus, the molecule-ion binding that occurs at air–water interfaces provides a well-defined environment to help understand design principles of interfacial recognition chemistry that cannot be achieved in the study of bulk soft-matter phases.^21,41,42^ Pioneering works from Kunitake^41,42^ and recent work from our groups^17–19^ have also shown that the air–water interface offers a lower dielectric constant that helps enhance affinity to offset the cost of anion dehydration upon binding. To the best of our knowledge, however, the triggered assembly of supra-amphiphiles into monolayers at the air–water interface have not been studied to help deconvolute the specificity of ion binding into the competing effects of recognition and hydration.

Herein, we study the structures, selectivities, and the driving forces of the anion-triggered self-organization of a supra-amphiphile at air–water interfaces. For this purpose, we tailored a cyanostar macrocycle, called cyanosurf, to generate surfactant properties upon anion binding. Cyanostar macrocycles show strong size-dependent binding ∼10^12^ M^−2^ in organic solvents with large and charge-diffused anions, such as PF_6_^−^ and ClO_4_^−^.^25^ Thus, we expect these hydrophobic anions to bind and trigger amphiphile formation leading to self-organization as a monolayer.^25^ To verify that binding is defined by the macrocycle's preprogrammed recognition properties, we compared the response to the smaller Cl^−^ anions, which has a low affinity for the macrocycle. To evaluate the role of hydration, we examined binding of H_2_PO_4_^−^. While this anion binds well to cyanostar in organic solutions,^43^ it has a high hydration energy. Thus, we set up a competition between the two driving forces of recognition and hydration. The interfacial binding and phase formation properties were studied using surface pressure–mean molecular area isotherms, Brewster angle microscopy (BAM) imaging of the surface, infrared reflection absorption spectroscopy (IRRAS), and sum frequency generation spectroscopy (SFG). These studies show that the cyanosurf molecules alone form an aggregate on pure water corresponding to the initially hydrophobic character of cyanosurf. Addition of PF_6_^−^ or ClO_4_^−^ anions in the subphase as sodium (Na^+^) salts initiates cyanosurf to self-organize into a well-ordered monolayer. Interfacial anion binding produces the supra-amphiphiles with the negatively charged cyanosurf-anion complex serving as aqueous anchors for monolayer formation. Addition of the hydrophilic Cl^−^ and H_2_PO_4_^−^ anions does not produce interfacial complexes. These studies show how interfacial binding and triggered monolayer formation are a balance between the pre-programmed molecular recognition and the anion's hydration properties. The combination of these factors is responsible for the binding of specific ions and needs to be considered in the design of supra-amphiphiles and extraction systems alike.

## Results and discussion

### Molecular design and synthesis

We designed an anion-triggered surfactant-forming receptor, cyanosurf, that is based on the cyanostar macrocycle. These macrocycles are also shape-persistent with well-defined and rigid structures and for this reason, the contribution of conformational flexibility to the binding and self-organization of the macrocyclic core can be neglected. This factor enables a more direct attribution of the resulting ion-triggered phase behavior to the intrinsic recognition properties of the macrocycle.

**Scheme 1:**
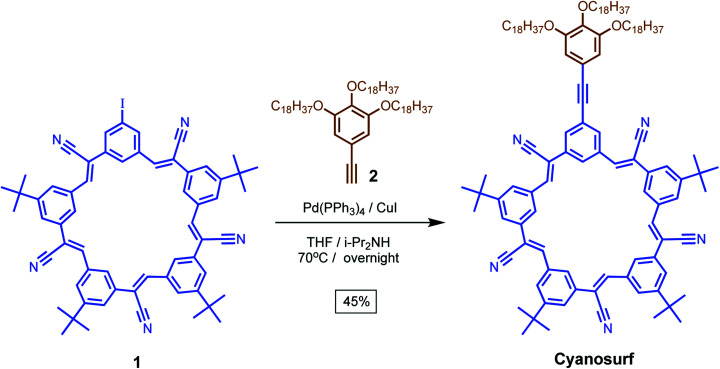
Synthesis of cyanosurf macrocycle.

To adapt this compound to supra-amphiphile formation, we incorporated three long octadecyl tails for interfacial anchoring. This target compound was made by coupling (Scheme 1) the iodo-cyanostar macrocycle, 1, with tris-alkoxy-5-ethynylbenzene, 2, under Sonogashira conditions in 45% yield according to previously reported procedures.^44,45^ Compound identity was confirmed using ^1^H, ^13^C{^1^H} NMR spectroscopy and high-resolution mass spectrometry.

### Self-association behavior in organic solution

**Fig. 2:**
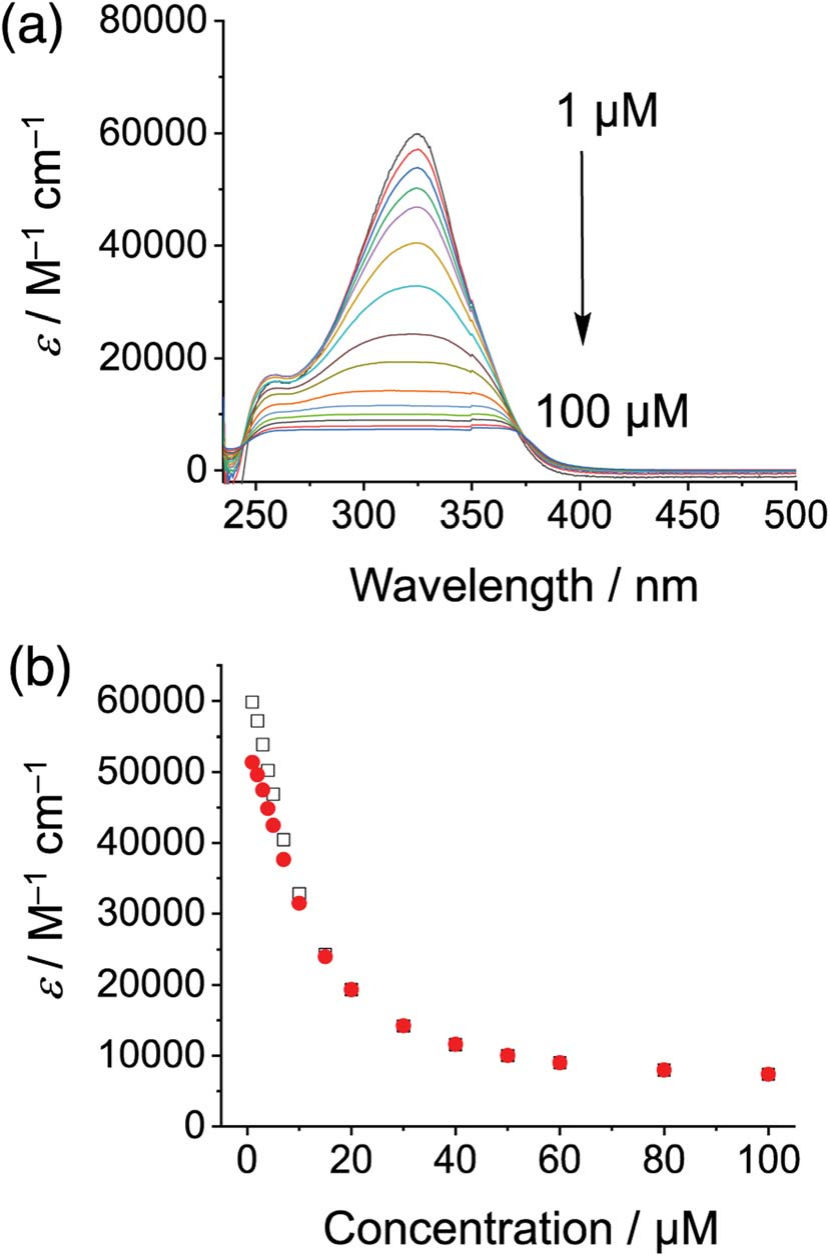
(a) Variable concentration studies of cyanosurf in organic solution observed using UV-Vis absorption (1–100 μM, CHCl_3_) normalized to the extinction coefficient and (b) analyzed according to an isodesmic model of self-association (325 nm white squares, 310 nm red circles).

Cyanosurf is expected and found to display similar properties as those of the parent cyanostar^25^ albeit with a greater propensity towards self-association. Variable concentration UV-Vis and NMR spectra are consistent with a high degree of self-association in chloroform. From 1 to 100 μM, the absorption spectra (Fig. 2a) show both substantial decreases in extinction coefficient from 60 000 to 8000 M^−1^ cm^−1^ with substantial peak broadening. The extent of broadening produces a flat-topped band suggesting that it might have been an artifact of the measurement. However, all the absorbance values were below 1.0. These effects are all typical of self-association.^46^ For this reason, the data was analyzed according to the simplest isodesmic model of self-association, so-called equal-K model^47^ (ESI†), and found to be consistent with a large self-association constant of over 10^5^ M^−1^.

**Fig. 3:**
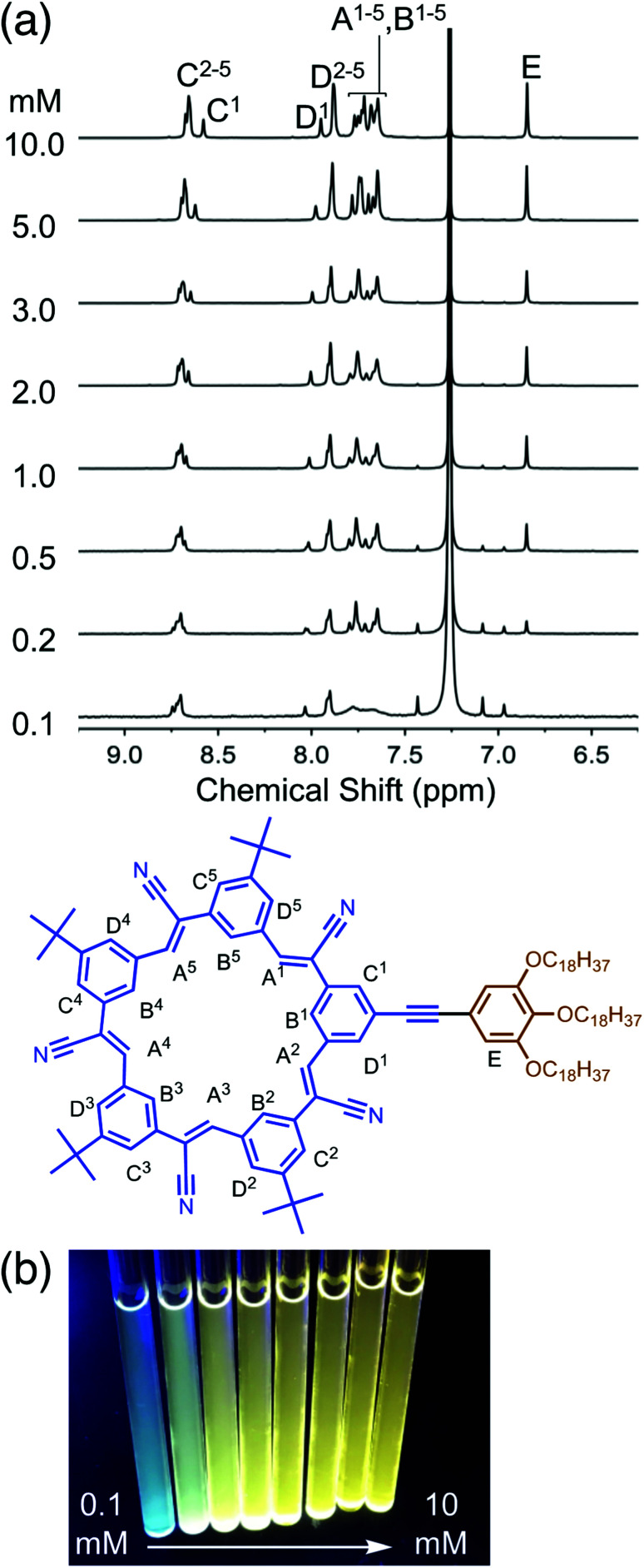
Variable concentration studies of cyanosurf in organic solution (a) observed using ^1^H NMR spectra recorded 0.1–10 mM (CDCl_3_, 298 K, 600 MHz) and (b) a photograph of the corresponding NMR tubes recorded under UV light illumination.

^1^H NMR spectroscopy (Fig. 3a) was used to provide insights into structural changes accompanying self-association. As the concentration is raised from 100 μM up another two orders of magnitude to 10 mM, the aromatic ^1^H NMR protons display upfield shifts indicative of π stacking.^46^ These shifts are seen in all the inner and outer cyanostar protons (H_A_, H_B_, H_C_, H_D_). Notably, however, the protons (H_E_) on the trialkoxy-substituted phenylene do not change position. This observation suggests that any association between neighboring molecules do not bring these pendant phenylenes into contact with each other. Across the same concentration range, we also observe (Fig. 3b) an increase in emission from the solution. This type of signature is often associated with the suite of phenomena broadly described as aggregation-induced emission.^48^ Taken together, these spectroscopies provide evidence that cyanosurf can support extensive self-association in organic chloroform solutions.

### Anion-binding triggered monolayer formation

**Fig. 4:**
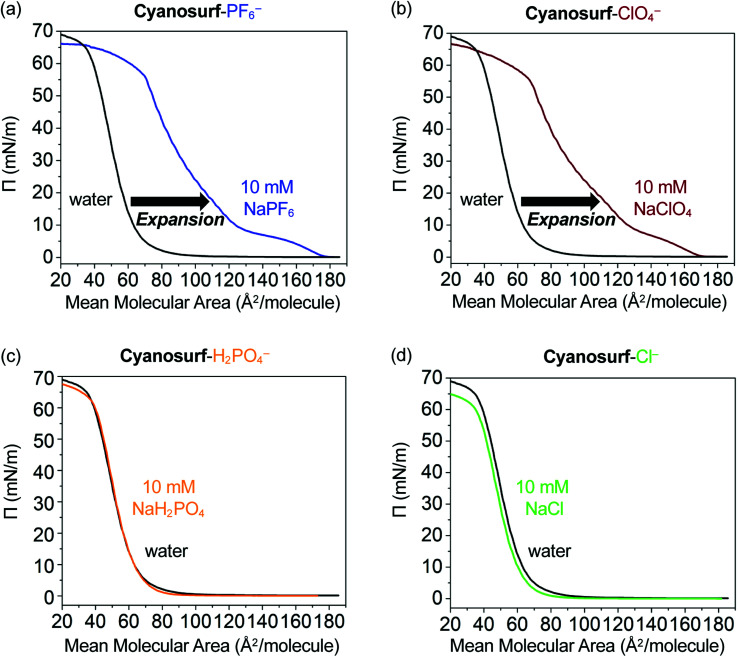
The *Π*–*A* isotherms of the cyanosurf molecules on water (black trace) and on a 10 mM aqueous solution of the sodium (Na^+^) salts of (a) hexafluorophosphate (PF_6_^−^) (blue trace), (b) perchlorate (ClO_4_^−^) (brown trace), (c) dihydrogen phosphate (H_2_PO_4_^−^) (orange trace), and (d) chloride (Cl^−^) (green trace), respectively.

The potential for interfacial anion binding leading to supra-amphiphile formation and subsequent surface self-assembly (Fig. 1b and c) of cyanosurf molecules with ClO_4_^−^, PF_6_^−^, H_2_PO_4_^−^ and Cl^−^ anions was investigated. Surface pressure–mean molecular area (*Π*–*A*, Fig. 4) isotherms were used to evaluate the interfacial recognition events between the neutral cyanosurf and aqueous anions. The samples were prepared by adding a known amount of the cyanosurf to the water phase as a chloroform solution and allowing the organic solvent to evaporate. Subsequently, *Π*–*A* isotherms of the cyanosurf deposited onto aqueous solution surfaces were recorded (black lines, Fig. 4) by compressing any surface-anchored species to smaller areas and measuring their growing surface pressures. The isotherms were also recorded with the cyanosurf deposited on solutions with 10 mM of PF_6_^−^ (blue line, Fig. 4a) and ClO_4_^−^ (brown line, Fig. 4b) in the aqueous solution. With these anions present, there is a significant expansion to larger mean molecular areas (MMAs) compared to the isotherm on pure water (black lines). With cyanosurf on aqueous solutions of PF_6_^−^, we see a mean molecular expansion from 65 to 122 Å^2^ per molecule (*Π* = 10 mN m^−1^) and a lift-off point above 170 Å^2^ per molecule. The isotherm of the cyanosurf molecule on ClO_4_^−^ also shows a similarly large expansion of the mean molecular area to 125 Å^2^ per molecule (*Π* = 10 mN m^−1^) with a lift-off point of ∼170 Å^2^ per molecule. Observation of larger mean molecular areas upon monolayer compression indicates that cyanosurf molecules display substantially different organization at the interface, which is consistent with the binding of PF_6_^−^ and ClO_4_^−^ anions to the cyanostar core seen in organic solution.^25^

The isotherms on PF_6_^−^ and ClO_4_^−^ show multiple phases. Lift-off is usually associated with the onset of a liquid-expanded phase. For both PF_6_^−^ and ClO_4_^−^, there also appears to be a turnover around 5 mN m^−1^ (150 Å^2^ per molecule) consistent with passing through a coexistence region containing both the liquid expanded and liquid condensed phases, and then a phase transition to the liquid condensed phase around 10 mN m^−1^. In the case of both anions, we see a second (∼10–20 mN m^−1^) and third (∼40–55 mN m^−1^) phase. These are usually associated with liquid-condensed phases having high and low tilting, respectively, and an intermediate coexistence region (∼20–40 mN m^−1^) in which both tilted phases exist. These transitions occur at slightly different pressures and MMAs for the two anions. Overall, both anions show a gradual nonlinear increase in surface pressure upon compression until a collapsed phase is produced at around 55 mN m^−1^ (70 Å^2^ per molecule). We attribute the behavior seen in the isotherm to formation of expanded and then condensed monolayers of the supra-amphiphiles.

Contrary to the significant expansion seen in the cases of PF_6_^−^ and ClO_4_^−^, the *Π*–*A* isotherms of cyanosurf with 10 mM of H_2_PO_4_^−^ or Cl^−^ present in bulk solution show a negligible change (Fig. 4c and d). This observation suggests that the interfacial arrangement of the cyanosurf molecules is unaltered when H_2_PO_4_^−^ and Cl^−^ are individually present in the aqueous solution. The negligible response to chloride is consistent with the selectivity preferences of the macrocycle,^25^ which disfavors smaller anions. However, phosphate is a size-matched anion that displays strong binding^49^ in organic solutions. On that basis alone, it is expected to display similar interfacial binding as PF_6_^−^ and ClO_4_^−^. However, the isotherm results indicate otherwise. To corroborate the results from the isotherm studies, aqueous surface imaging was undertaken.

### Surface organization by Brewster angle imaging

**Fig. 5:**
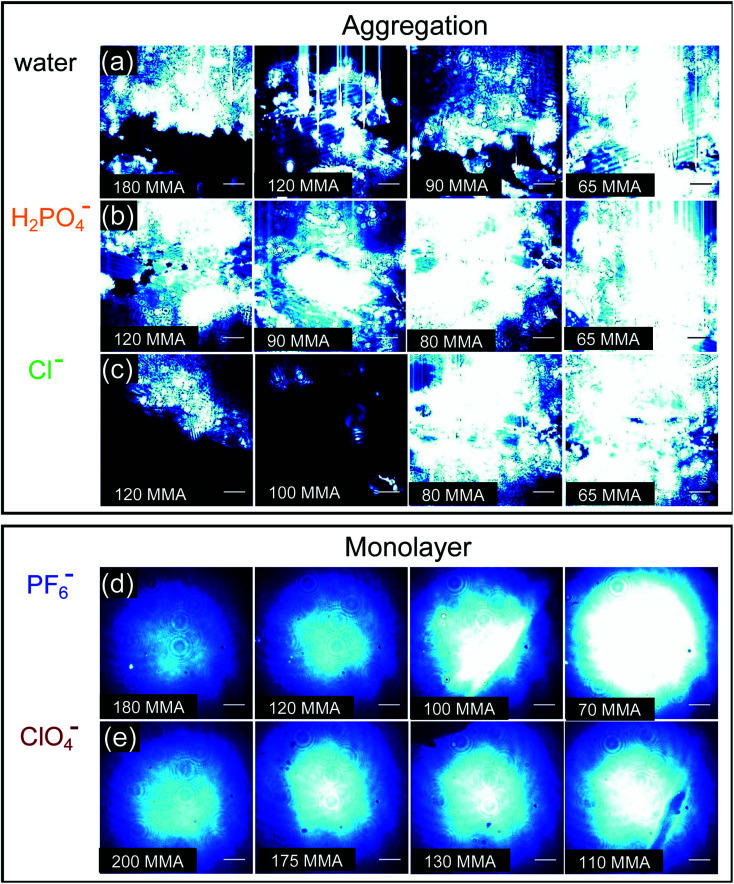
BAM images corresponding to the compression of the cyanosurf molecules on (a) H_2_O and the aqueous solutions of the sodium salts at 10 mM of (b) Cl^−^, (c) H_2_PO_4_^−^ (d) PF_6_^−^, and (e) ClO_4_^−^, respectively. Images (a)–(e) correspond to different mean molecular areas (MMAs) to show the range of the *Π*–*A* isotherms. The blue color in the image is artificial for better contrast; the beam focus gives rise to the central brightness of (d) and (e). The scale bar is 50 μm.

BAM images provide real-time ∼1 μm resolution aqueous surface imaging of the morphological changes in the packing structure of the cyanosurf molecules throughout the *Π*–*A* isotherm. The bright areas of the images correspond to regions enriched in molecular species (non-solvent molecules) at the surface and the dark areas are poor in surface active molecules (and are therefore water-rich). The increase in brightness scales with changes in refractive index relative to the bulk solution. For this reason, the brighter areas of the image correspond to either more dense and/or assemblies of molecules enriched at the surface that extend into three-dimensions (3D). Consequently, 2D monomolecular monolayer films are less bright compared to surface-aggregates that are 3D. It is evident from the BAM imaging that there are significant amounts of 3D structures with cyanosurf on water, on phosphate, and on the chloride solutions with the brightness increasing upon compression of the monolayer (Fig. 5a–c). The 3D structures begin to form when the cyanosurf molecules are at low average film density with MMAs = 180, 120, and 120 Å^2^ per molecule for pure water, H_2_PO_4_^−^ and Cl^−^, respectively, even though the surface pressure has yet to lift off at *Π* = 0 mN m^−1^. The observation of 3D structures (*i.e.*, aggregates) of the cyanosurf macrocycle at the interface is consistent with the self-association phenomena for shape-persistent macrocycles in bulk solution.^46,50,51^ Thus, the cyanosurf molecules exist in an aggregated form in chloroform prior to deposition and retain that form on the surface of water. According to the BAM study, the presence of H_2_PO_4_^−^ and Cl^−^ anions in the aqueous solution are not able to induce a re-organization of the aggregated cyanosurf molecules into an ordered monolayer through interfacial binding.

In comparison, we observed neat and homogeneous film formation when cyanosurf was deposited on aqueous solutions of PF_6_^−^ and ClO_4_^−^. BAM imaging shows (Fig. 5d) a homogeneous cyanosurf monolayer is formed with PF_6_^−^ in the expanded region at 180 Å^2^ per molecule in stark contrast to bright 3D structures observed with water, phosphate, and chloride. The molecular density increases upon compression and we see increasing brightness for the neat film (Fig. 5d). We observe a similar result for cyanosurf on ClO_4_^−^ solutions (Fig. 5e). Clearly, addition of PF_6_^−^ and ClO_4_^−^ drives 2D monolayer film organization. The only mechanism by which this occurs is for transformation of the hydrophobic macrocycle into a supra-amphiphilic complex upon anion binding (Fig. 1b). Thus, the charged complex orients into the aqueous subsurface while the alkyl chains are directed towards the air side of the aqueous interface to help with film organization.

### Molecular model of the anion complexed supra-amphiphile

**Fig. 6:**
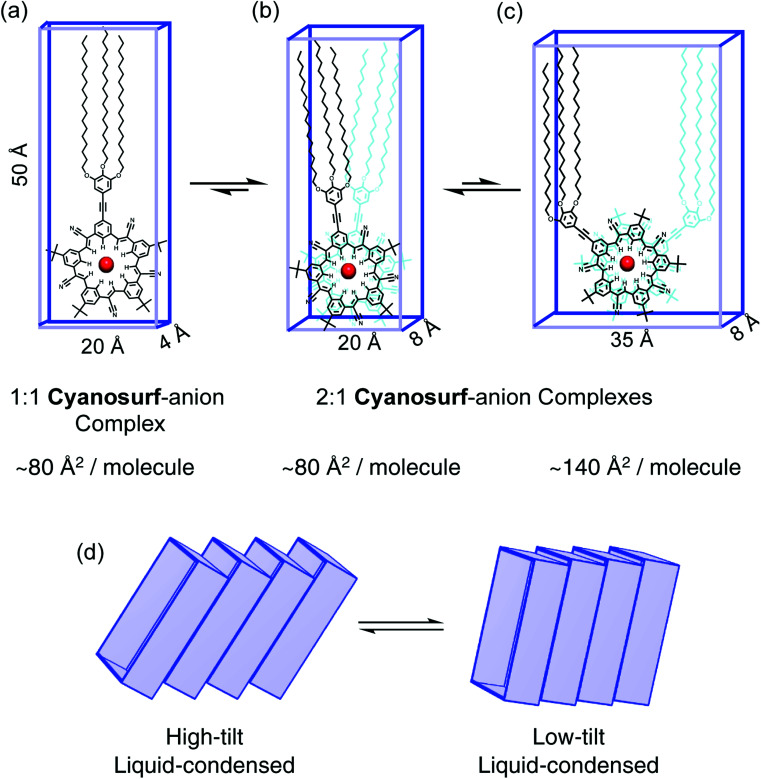
Molecular models of the supra-amphiphile complexes at the air–water interface as (a) a 1 : 1 complex, (b) 2 : 1 complexes with either ∼36° or (c) ∼108° rotation angles that are defined by the sterically-geared *tert*-butyl groups on the macrocycles. (d) Tilted arrangements of the supra-amphiphiles.

Models of the possible amphiphilic complexes (Fig. 6) that can self-assemble upon anion binding at the interface are proposed. Under the conditions of the experiment there is an excess of anions (ClO_4_^−^, PF_6_^−^) in the subphase. Excess anion typically favors 1 : 1 binding stoichiometries (Fig. 6a). In the case of the cyanostar macrocycles with ClO_4_^−^ and PF_6_^−^, however, they prefer 2 : 1 complexation by forming a π-stacked seam (Fig. 6b). We also know that the steric gearing between *tert*-butyl substituents on the π-stacked macrocycles predefines a limited number of macrocycle–macrocycle rotational angles at ∼36° (Fig. 6b) or 108° (Fig. 6c) relative to each other.^52^ An angle of 180° is also possible but would lead to bolaphiles with the large trialkoxy chains at opposite ends of the complex. Modelling shows that the smaller angle (36°) has the most efficient packing of space. The six alkoxy chains are better size-matched to the ∼20 Å × 8 Å footprint of the cyanosurf-anion complex of the hydrophilic group. The fact that this complex involves two macrocycles, it defines a cross-sectional area of 80 Å^2^ per molecule, which is the MMA value approached at higher surface pressures for PF_6_^−^ and ClO_4_^−^ (Fig. 4). While this MMA is also similar to the surface density of the 1 : 1 complex (Fig. 6a), the 2 : 1 complexes are favored by these anions.

The observation of larger MMAs seen initially, *e.g.*, ∼170 Å^2^ per molecule at lift-off, suggest that different arrangements are possible. These include a different rotation angle (108°) with larger MMA (Fig. 6c) or tilting (Fig. 6d) could exist through the liquid-expanded phases. In the putative liquid-expanded and co-existence regions, the 2 : 1 complexes may have a range of local structures. These include mixtures of the 36° and 108° rotational complexes as well as a variation in the tilt angles. At the high-density liquid-condensed region generated under high compression (*Π* = 10–55 mN m^−1^), however, the 2 : 1 stoichiometries favored by the anion-recognition properties of cyanostar^25^ are likely to produce 36° rotations between the two complexed macrocycles.

### Surface spectroscopy of the interfacial anion-bound complexes

**Fig. 7:**
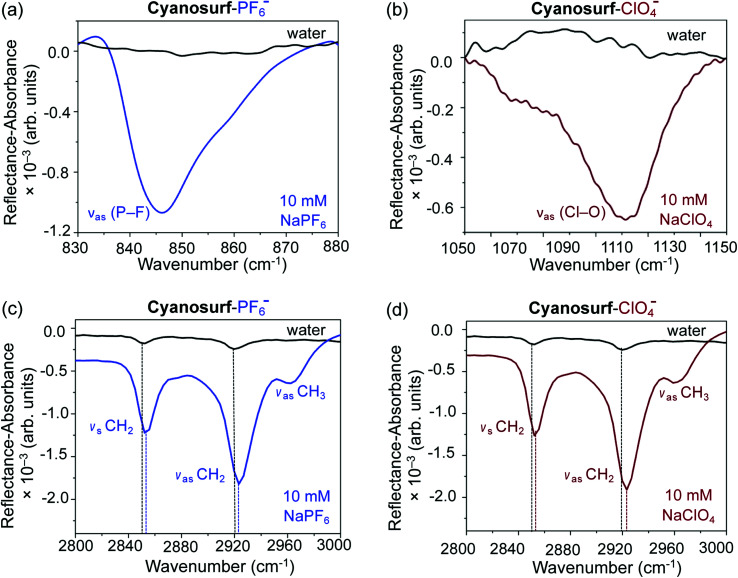
IRRAS spectra showing cyanosurf reorganization upon anion binding. The spectrum of the cyanosurf molecules on water is shown as a black trace for (a)–(d). The spectra of the cyanosurf molecules with 10 mM of the sodium salts of (a) and (c) ClO_4_^−^ (brown) and (b) and (d) PF_6_^−^ (blue) at a MMA of 150 Å^2^ per molecule.

In order to better understand some of the molecule-level details of the binding events occurring between cyanosurf and anions at the interface, spectroscopic studies using infrared reflection absorption spectroscopy (IRRAS) were performed. The IRRAS data was collected at 150 Å^2^ per molecule coinciding with the liquid expanded region that shows a highly ordered monolayer by BAM analysis and is assigned to formation of the 2 : 1 supra-amphiphile complex. The IRRAS data are plotted as reflectance–absorbance (RA) spectra, which is given as RA = −log(*R*_c_/*R*_0_), where *R*_c_ is the reflectivity of the cyanosurf surface and *R*_0_ is the reflectivity of the subphase solution, *i.e.*, either water, PF_6_^−^ or ClO_4_^−^ aqueous solutions. On account of the fact that the spectra of the PF_6_^−^ or ClO_4_^−^ subphase solution is present in both the numerator and denominator leads to IRRAS signals being sensitive solely to the cyanosurf-bound ions. Downward peaks are observed, as expected for reflectivity studies in this optical geometry.^53^ There are clear vibrational signatures from perchlorate and hexafluorophosphate anions bound to the cyanosurf molecules in the monolayers. Broad peaks appear for cyanosurf-PF_6_^−^ (Fig. 7a) and cyanosurf-ClO_4_^−^ (Fig. 7b) at approximately 846 and 1110 cm^−1^, and are assigned to the hexafluorophosphate antisymmetric (*ν*_as_ P–F) and perchlorate asymmetric (*ν*_as_ Cl–O) stretching modes, respectively.^54,55^ The presence of these peaks supports the binding of PF_6_^−^ and ClO_4_^−^ to the cyanosurf molecules. Similar anion vibrational signatures for phosphate in the presence of the cyanosurf molecules are absent (ESI, Fig. S4†).

The alkyl stretching region provides useful insight into the molecular organization within the monolayer. With cyanosurf on water (black traces, Fig. 7c and d), the IRRAS spectra show two distinct albeit weak peaks at 2852 and 2920 cm^−1^ that are assigned to the CH_2_ symmetric and asymmetric stretches, respectively. When comparing these alkyl modes to those of the cyanosurf on aqueous solutions of PF_6_^−^ and ClO_4_^−^, the intensities of both peaks have increased substantially and are blue shifted (Fig. 7c and d). A third peak at 2963 cm^−1^ also becomes more prominent for cyanosurf on PF_6_^−^ and ClO_4_^−^ and is assigned to the asymmetric stretch of the terminal CH_3_ groups. We attribute the observed changes in intensity to the transition from 3D aggregates of the cyanosurf to a monolayer of the cyanosurf-anion complex seen by BAM imaging. The increase in the IRRAS intensity of the vibrations associated with the anions and the CH modes of the alkyl chains that occur with anion complexation indicates that interfacial complexation of the anions and re-organization into a homogeneous monolayer film. For H_2_PO_4_^−^ and Cl^−^ solutions, we observe a substantial loss of the cyanosurf methylene signature compared to that on water (ESI†), in opposition to what is observed from cyanosurf on PF_6_^−^ and ClO_4_^−^ solutions. This observation is consistent with the picture of anion-triggered cyanosurf surface complexation with PF_6_^−^ and ClO_4_^−^ and not with the H_2_PO_4_^−^ and Cl^−^ anions.

As seen in the BAM imaging, cyanosurf prefers to retain an aggregated form on water and is not distributed as a homogenous monolayer. In this situation, the average spectra recorded using IRRAS is a combination of mostly the bare water surface and any of the 3D aggregated structures of cyanosurf. This averaging produces the weak signature of the hydrocarbon chains observed in the IRRAS spectra. However, in the presence of the PF_6_^−^ and ClO_4_^−^, the cyanosurf molecules bind to these anions likely as a 2 : 1 sandwich complex (Fig. 6b and c) and self-organize into the monolayer (Fig. 1c). With the cyanosurf macrocycles bound to the anions on the surface, the hydrocarbon chains organize towards the air side of the interface. This anion-driven transition of the cyanosurf-aggregates (solid phase, 3D aggregates) to the cyanosurf-monolayer (amphiphilic phase) gives rise to a blue shift of the *ν*_s_ CH_2_ and *ν*_as_ CH_2_ stretching modes. The alkyl chains in the aggregated phase are seen to vibrate at lower frequencies indicating that either some or all of the chains are organized in the all-*trans* methylene–methylene conformations. These conformations facilitate packing and thus intermolecular interactions between the adjacent chains. Transformations from all *trans* to *gauche* are commonly observed with infrared frequency shifts.^53,56^ More *gauche* defects are consistent with higher methylene stretch frequencies (blue shift). Additional *gauche* defects can be accommodated in the amphiphilic monolayer when there is more space available for the chains and when organization depends less on the inter-alkyl interactions. In the monolayer arrangement, it is clear that the anion-bound complex possesses a larger footprint than its six tails alone and the resultant structure allows room for some disorder in the chains. Therefore, the charged macrocyclic headgroup of the cyanosurf complexes likely plays a larger role than the tails on ordering the monolayer.

### Spectroscopy of the interfacial hydration layer defined by the cyanosurf-anion monolayer

**Fig. 8:**
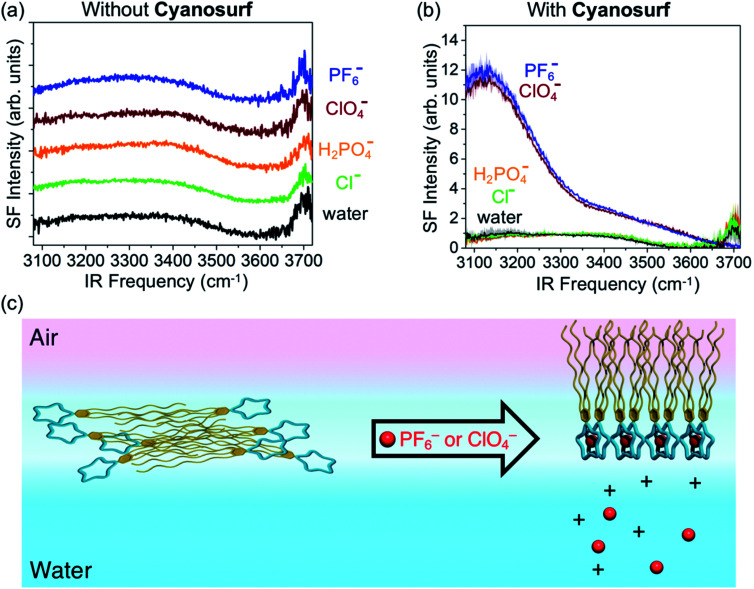
Sum frequency generation spectra of the: (a) H_2_O, Cl^−^, H_2_PO_4_^−^, PF_6_^−^, and ClO_4_^−^ aqueous solutions without the cyanosurf shown with a baseline offset for clarity, (b) and with the cyanosurf. (c) Schematic representation of the anion-induced organization of the cyanosurf monolayer. Spectra were obtained after an equilibration time of 10 minutes following the spreading of cyanosurf to a constant MMA of 150 Å^2^ per molecule.

To investigate the surface hydration and water alignment effects that are induced by the organized cyanosurf-anion monolayer in the liquid-expanded region, we used sum frequency generation (SFG) spectroscopy. This technique selectively probes the topmost layers of aqueous surfaces. In addition, the presence of charged interfaces greatly enhances the depth that contributes to the SFG signal. The spectra were taken in the ssp polarization combination (s-SFG, s-visible, p-infrared) in the region from 3800 to 3000 cm^−1^. SFG spectra of neat water and 10 mM salt solutions were recorded first in the absence of cyanosurf (Fig. 8a). The neat water spectra (black trace) all consist of a broad band below 3600 cm^−1^ corresponding to the hydrogen-bonded water and a sharp peak centered at ∼3700 cm^−1^ assigned to dangling OH bonds from the topmost layer of water.

Recent SFG studies from the surfaces of low-concentration salt solutions show that the intensity of the lower frequency OH stretch region (<3300 cm^−1^) corresponds to water molecules in sub-surface layers. As a consequence, the signal intensity stems from the bulk *χ*^(3)^ contribution arising from an electric double layer.^57–59^ In this situation, the transition dipole moments of the water molecules align normal to the surface and result in an increase in the SFG intensity at ∼3200 cm^−1^. We do not observe any such changes in the SFG spectra upon addition of the salt at 10 mM concentration without the cyanosurf. Therefore, use of such low salt concentrations produce very little change in the organization of surface water.

SFG spectra recorded in the presence of the cyanosurf molecules show dramatic changes upon selective anion binding (Fig. 8b). First, the SFG spectra for cyanosurf on water, chloride and phosphate solutions show similar features when compared to the SFG spectra recorded without the cyanosurf (Fig. 8a). This similarity reinforces our observations that there is little interaction between the cyanosurf receptor and Cl^−^ or H_2_PO_4_^−^ anions.

For the liquid-expanded phase composed of the supra-amphiphilic 2 : 1 cyanosurf-anion complexes on PF_6_^−^ and ClO_4_^−^ aqueous solutions (Fig. 8b), however, we observe a dramatic enhancement in the intensities of “OH” stretching modes for water at ∼3150 cm^−1^. This enhancement is attributed to the *χ*^(3)^ effect, which is also termed the *χ*^(2)^ diffuse layer (*χ*^(2)^_DL_),^60^ and is associated with the alignment of water stemming from large electrostatic fields. With the addition of PF_6_^−^ and ClO_4_^−^, this enhancement is more than 10 times higher when compared to all other spectra. The electrostatic field that aligns the water molecules and gives rise to the enhanced SFG signal is a result of the self-organization of the amphiphilic cyanosurf-anion complexes into charged monolayers at the surface of water. Alignment of the surface and subsurface water molecules thus removes the solution centrosymmetry allowing more water molecules to contribute to the nonlinear SFG polarization response. This behavior is consistent with reports by several groups of charged surfactant–water interfaces.^61–63^

### Chemical and physical contributions to the driving forces of interfacial anion recognition

The selective response of cyanosurf to PF_6_^−^ and ClO_4_^−^ but not Cl^−^ follows from the size-dependent binding dictated by the macrocyclic core but does not explain the response to phosphate. This anion has been shown to form oligomers that recruit as many as four cyanostar macrocycles into a large assembly^49^ with binding energies on par with PF_6_^−^ and ClO_4_^−^. Yet, by all measures, we see a negligible molecular recognition response to the H_2_PO_4_^−^ anion. This anomaly can be explained by taking into account the fact that H_2_PO_4_^−^ anions are highly hydrated (−465 kJ mol^−1^).^64,65^ By comparison, the lower dehydration penalties are easier to pay in the case of the less hydrated PF_6_^−^ and ClO_4_^−^ anions (−71 and −205 kJ mol^−1^, respectively).

The observation that hydration energies play a role is consistent with prior work revealing that positively charged receptors are key to driving interfacial phosphate binding through electrostatics-assisted hydrogen bonding.^17^ Clearly the neutral cyanostar macrocycle's binding energy is not able to offset the high hydration energy of the H_2_PO_4_^−^ anion. That prior work also showed that the interfacial phosphate binding energies were enhanced on order 10 000× over bulk aqueous solution. To provide a measure of the affinity, we measured the binding of ClO_4_^−^ to the monolayer. We used a MMA of 150 Å^2^ per molecule and fit the data to a 2 : 1 binding model to obtain a value of 1000 M^−1^. We cannot compare this value to aqueous solution because cyanosurf or any of its homologs are not soluble in water. The only comparison we have is to MeOH–CHCl_3_ solutions where affinity is as high as 10^12^ M^−1^. We attribute the fall-off in stability to the steep cost of dehydration.

## Conclusion

We provide the first account of supra-amphiphilic film formation based on anion-selective recognition and its use to understand the driving forces that govern interfacial anion binding. The cyanosurf molecules are initially aggregated, however, upon binding perchlorate (ClO_4_^−^) and hexafluorophosphate (PF_6_^−^) from aqueous solutions they become organized as monolayers. Without the binding, the cyanosurf molecules remain in an aggregated state on pure water and on phosphate and chloride aqueous subphases. For the PF_6_^−^ and ClO_4_^−^ anions, surface pressure isotherms reveal large expansion signatures with well-behaved phases. Aqueous surface microscopy shows production of an ordered amphiphilic 2D monolayer phase. Infrared reflectivity studies show vibrational spectra of surface anion modes indicative of their complexation and a reduction in chain organization indicating the charged headgroup dictates monolayer ordering. Vibrational sum frequency generation spectroscopy reveals that the monolayer formed upon cyanosurf-mediated binding of ClO_4_^−^ and PF_6_^−^ produces a substantial negatively-charged interface that aligns water molecules perpendicular to the surface plane. The same outcomes are not present with Cl^−^ and H_2_PO_4_^−^. Chemical selectivity is conferred on the interfacial molecular recognition by the size-dependent binding of cyanosurf to ClO_4_^−^ and PF_6_^−^ anions. Physical selectivity is conferred on the interfacial recognition by the hydration energy penalty that disfavors phosphate. Chloride binding is disfavored on chemical and physical grounds. Phosphate binding is disfavored on physical grounds alone. The study of anion binding induced re-organization of the cyanosurf monolayer helps deepen our understanding of molecule-mediated recognition phenomena that trigger formation of soft-matter phases at aqueous interfaces.

## Author contributions

L. Y., A. S., W. Z., J. F. N. and Y. C. conducted the studies and data analysis. L. Y. and W. Z. wrote the original draft. A. H. F. and H. C. A. conceptualized and supervised the research. All authors contributed to the manuscript writing and editing.

## Conflicts of interest

The authors declare no competing financial interest.

## Supplementary Material

SC-013-D2SC00986B-s001

## References

[cit1] Kimizuka N. (2003). Curr. Opin. Chem. Biol..

[cit2] Zhang X., Wang C. (2011). Chem. Soc. Rev..

[cit3] Wang C., Wang Z., Zhang X. (2012). Acc. Chem. Res..

[cit4] Liu K., Kang Y. T., Wang Z. Q., Zhang X. (2013). Adv. Mater..

[cit5] Fu S., Su X., Li M., Song S. L., Wang L., Wang D., Tang B. Z. (2020). Adv. Sci..

[cit6] Hu X. Y., Liu X., Zhang W. Y., Qin S., Yao C. H., Li Y., Cao D. R., Peng L. M., Wang L. Y. (2016). Chem. Mater..

[cit7] Xu X. H., Li Y. K., Li H. P., Liu R., Sheng M. M., He B., Gu Z. W. (2014). Small.

[cit8] Li J. X., Wang Z. R., Zhou J., Li M. J., Luo Q., Dong Z. Y., Shi S., Liu J. Q. (2018). Colloids Surf., A.

[cit9] Chiarizia R., Jensen M. P., Rickert P. G., Kolarik Z., Borkowski M., Thiyagarajan P. (2004). Langmuir.

[cit10] Wintergerst M. P., Levitskaia T. G., Moyer B. A., Sessler J. L., Delmau L. H. (2008). J. Am. Chem. Soc..

[cit11] Mu J. J., Motokawa R., Akutsu K., Nishitsuji S., Masters A. J. (2018). J. Phys. Chem. B.

[cit12] Liu Y. Y., Gao Y., Wei Z., Zhou Y., Zhang M., Hou H. G., Tian G. X., He H. (2018). J. Radioanal. Nucl. Chem..

[cit13] Cheisson T., Schelter E. J. (2019). Science.

[cit14] Nayak S., Lovering K., Uysal A. (2020). Nanoscale.

[cit15] Nayak S., Kumal R. R., Uysal A. (2021). ChemRxiv.

[cit16] Lovering K., Nayak S., Bu W., Uysal A. (2020). J. Phys. Chem. C.

[cit17] Neal J. F., Zhao W., Grooms A. J., Flood A. H., Allen H. C. (2018). J. Phys. Chem. C.

[cit18] Neal J. F., Zhao W., Grooms A. J., Smeltzer M. A., Shook B. M., Flood A. H., Allen H. C. (2019). J. Am. Chem. Soc..

[cit19] Grooms A. J., Neal J. F., Ng K. C., Zhao W., Flood A. H., Allen H. C. (2020). J. Phys. Chem. A.

[cit20] Neal J. F., Saha A., Zerkle M. M., Zhao W., Rogers M. M., Flood A. H., Allen H. C. (2020). J. Phys. Chem. A.

[cit21] Sakurai M., Tamagawa H., Inoue Y., Ariga K., Kunitake T. (1997). J. Phys. Chem. B.

[cit22] Cremer P. S., Flood A. H., Gibb B. C., Mobley D. L. (2018). Nat. Chem..

[cit23] Judd K. D., Gonzalez N. M., Yang T. L., Cremer P. S. (2022). J. Phys. Chem. Lett..

[cit24] Ariga K. (2020). Langmuir.

[cit25] Lee S., Chen C. H., Flood A. H. (2013). Nat. Chem..

[cit26] Thevenet A., Miljkovic A., La Cognata S., Marie C., Tamain C., Boubals N., Mangano C., Amendola V., Guilbaud P. (2021). Dalton Trans..

[cit27] Huang F. H., Zhang X. (2020). Mater. Chem. Front..

[cit28] Kang Y. T., Tang X. Y., Cai Z. G., Zhang X. (2016). Adv. Funct. Mater..

[cit29] Jeon Y. J., Bharadwaj P. K., Choi S., Lee J. W., Kim K. (2002). Angew. Chem., Int. Ed..

[cit30] Yu G. C., Zhou X. R., Zhang Z. B., Han C. Y., Mao Z. W., Gao C. Y., Huang F. H. (2012). J. Am. Chem. Soc..

[cit31] Chi X., Peters G. M., Hammel F., Brockman C., Sessler J. L. (2017). J. Am. Chem. Soc..

[cit32] Xia D. Y., Wang P., Shi B. B. (2017). Org. Lett..

[cit33] Jarvie H. P., Sharpley A. N., Flaten D., Kleinman P. J. A., Jenkins A., Simmons T. (2015). J. Environ. Qual..

[cit34] Tilman D., Balzer C., Hill J., Befort B. L. (2011). Proc. Natl. Acad. Sci. U. S. A..

[cit35] Hepburn C., Adlen E., Beddington J., Carter E. A., Fuss S., Mac Dowell N., Minx J. C., Smith P., Williams C. K. (2019). Nature.

[cit36] National Academies of Sciences, Engineering, and Medicine , A Research Strategy for Ocean-based Carbon Dioxide Removal and Sequestration, The National Academies Press, Washington, DC, 2021, 10.17226/26278 35533244

[cit37] He Q., Ao Y. F., Huang Z. T., Wang D. X. (2015). Angew. Chem., Int. Ed..

[cit38] Chi X. D., Peters G. M., Brockman C., Lynch V. M., Sessler J. L. (2018). J. Am. Chem. Soc..

[cit39] Jie K. C., Zhou Y. J., Yao Y., Huang F. H. (2015). Chem. Soc. Rev..

[cit40] Chaikittisilp W., Yamauchi Y., Ariga K. (2022). Adv. Mater..

[cit41] Onda M., Yoshihara K., Koyano H., Ariga K., Kunitake T. (1996). J. Am. Chem. Soc..

[cit42] Sasaki D. Y., Kurihara K., Kunitake T. (1991). J. Am. Chem. Soc..

[cit43] Fatila E. M., Pink M., Twum E. B., Karty J. A., Flood A. H. (2018). Chem. Sci..

[cit44] Zhu C., Wang T.-H., Su C.-J., Lee S.-L., Rives A., Duhayon C., Kauffmann B., Maraval V., Chen C.-h., Hsu H.-F. (2017). Chem. Commun..

[cit45] Benson C. R., Maffeo C., Fatila E. M., Liu Y., Sheetz E. G., Aksimentiev A., Singharoy A., Flood A. H. (2018). Proc. Natl. Acad. Sci. U. S. A..

[cit46] Lee S., Hirsch B. E., Liu Y., Dobscha J. R., Burke D. W., Tait S. L., Flood A. H. (2016). Chem.–Eur. J..

[cit47] Martin R. B. (1996). Chem. Rev..

[cit48] Hong Y., Lam J. W. Y., Tang B. Z. (2011). Chem. Soc. Rev..

[cit49] Fatila E. M., Pink M., Twum E. B., Karty J. A., Flood A. H. (2018). Chem. Sci..

[cit50] Zhang W., Moore J. S. (2006). Angew. Chem., Int. Ed..

[cit51] Wang Q., Zhong Y., Miller D. P., Lu X., Tang Q., Lu Z.-L., Zurek E., Liu R., Gong B. (2020). J. Am. Chem. Soc..

[cit52] Liu Y., Singharoy A., Mayne C. G., Sengupta A., Raghavachari K., Schulten K., Flood A. H. (2016). J. Am. Chem. Soc..

[cit53] Mendelsohn R., Brauner J. W., Gericke A. (1995). Annu. Rev. Phys. Chem..

[cit54] Heyns A. (1977). Spectrochim. Acta, Part A.

[cit55] Chen Y., Zhang Y.-H., Zhao L.-J. (2004). Phys. Chem. Chem. Phys..

[cit56] Adams E. M., Verreault D., Jayarathne T., Cochran R. E., Stone E. A., Allen H. C. (2016). Phys. Chem. Chem. Phys..

[cit57] Ohno P. E., Wang H.-f., Paesani F., Skinner J. L., Geiger F. M. (2018). J. Phys. Chem. A.

[cit58] Reddy S. K., Thiraux R., Wellen Rudd B. A., Lin L., Adel T., Joutsuka T., Geiger F. M., Allen H. C., Morita A., Paesani F. (2018). Chem.

[cit59] Ishiyama T., Shirai S., Okumura T., Morita A. (2018). J. Chem. Phys..

[cit60] Pezzotti S., Galimberti D. R., Shen Y. R., Gaigeot M. P. (2018). Phys. Chem. Chem. Phys..

[cit61] Gragson D., McCarty B., Richmond G. (1997). J. Am. Chem. Soc..

[cit62] Gragson D., Richmond G. (1997). J. Chem. Phys..

[cit63] Miranda P., Du Q., Shen Y. (1998). Chem. Phys. Lett..

[cit64] Smith D. W. (1977). J. Chem. Educ..

[cit65] Collins K. D. (2006). Biophys. Chem..

