# Supplementary material for: Recognition competes with hydration in anion-triggered monolayer formation of cyanostar supra-amphiphiles at aqueous interfaces
Source: Chem Sci. 2022 Mar 15;13(15):4283–94. doi: 10.1039/d2sc00986b (PMC9006960; doi:10.1039/d2sc00986b)
Supplement: SC-013-D2SC00986B-s001 [file SC-013-D2SC00986B-s001.pdf]

Electronic Supplementary Material (ESI) for Chemical Science.
This journal is © The Royal Society of Chemistry 2022

Electronic Supplementary Information

Recognition competes with hydration in anion-triggered monolayer formation of cyanostar supra-amphiphiles at aqueous interfaces

Liwei Yan [§†], Ankur Saha[§†], Wei Zhao [§‡], Jennifer F. Neal[†], Yusheng Chen[‡], Amar H. Flood[*‡], Heather C. Allen[*†]

General Methods

Reagents were obtained from commercial suppliers and used as received unless otherwise indicated. Column chromatography was performed on silica gel (160–200 mesh, Sorbent Technologies, USA). Thin-layer chromatography (TLC) was performed on pre-coated silica gel plates (0.25 mm thick) and observed under UV light. Nuclear magnetic resonance (NMR) spectra were recorded on Varian Inova (500 MHz and 400 MHz) spectrometers at room temperature (298 K). Chemical shifts were referenced to residual solvent peaks. The UV-Vis spectra were recorded on a Varian Cary 5000 UV-Vis-NIR spectrophotometer. The iodo-cyanostar macrocycle **1** and tris-alkoxy-5-ethynylbenzene precursor **2** (Scheme S1) were synthesized according to previously reported procedure[S1, S2] and confirmed based on the ^{1}H NMR spectra by comparing to the reported.

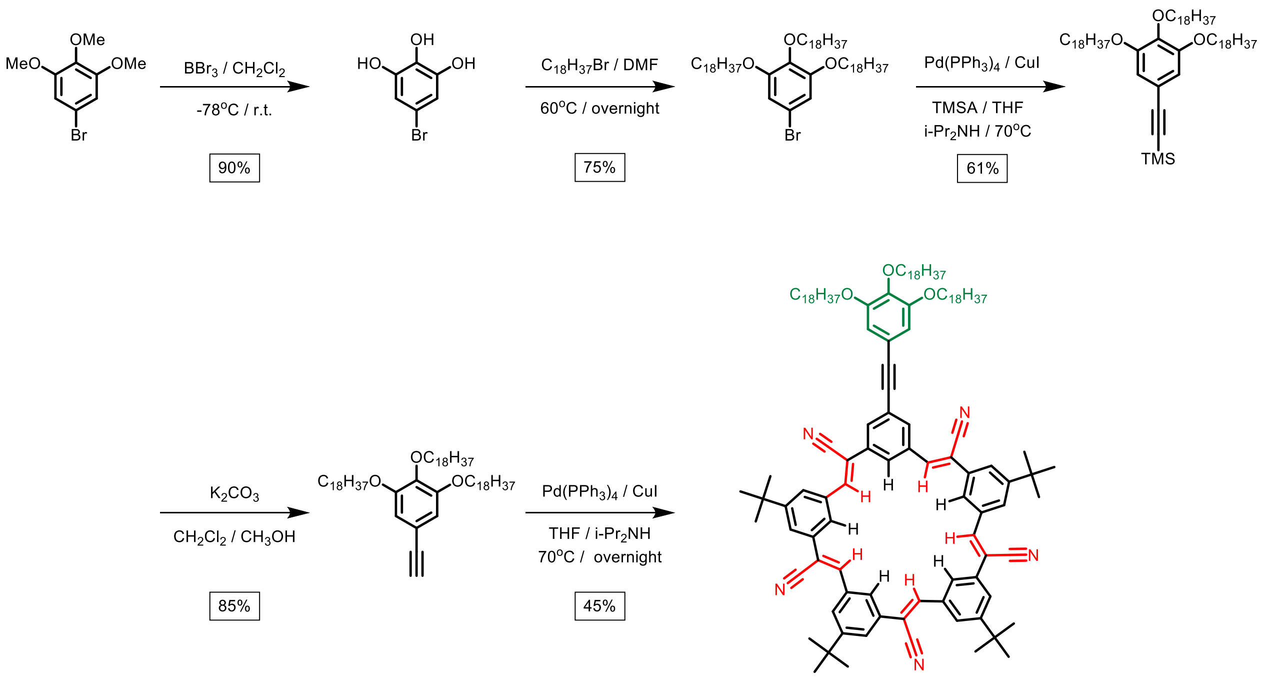

Scheme S1. Full synthetic route for **cyanosurf** macrocycle **3**.

^1H NMR (400 MHz, $CDCl_3$) of the iodo-cyanostar macrocycle **1**: δ = 8.84 (s, 1H), 8.67-8.63 (m, 4H), 8.14 (s, 1H), 7.88-7.85 (m, 4H), 7.75 (s, 1H), 7.72-7.70 (m, 4H), 7.63-7.61 (m, 4H), 7.58 (s, 1H), 1.47 (s, 36H).

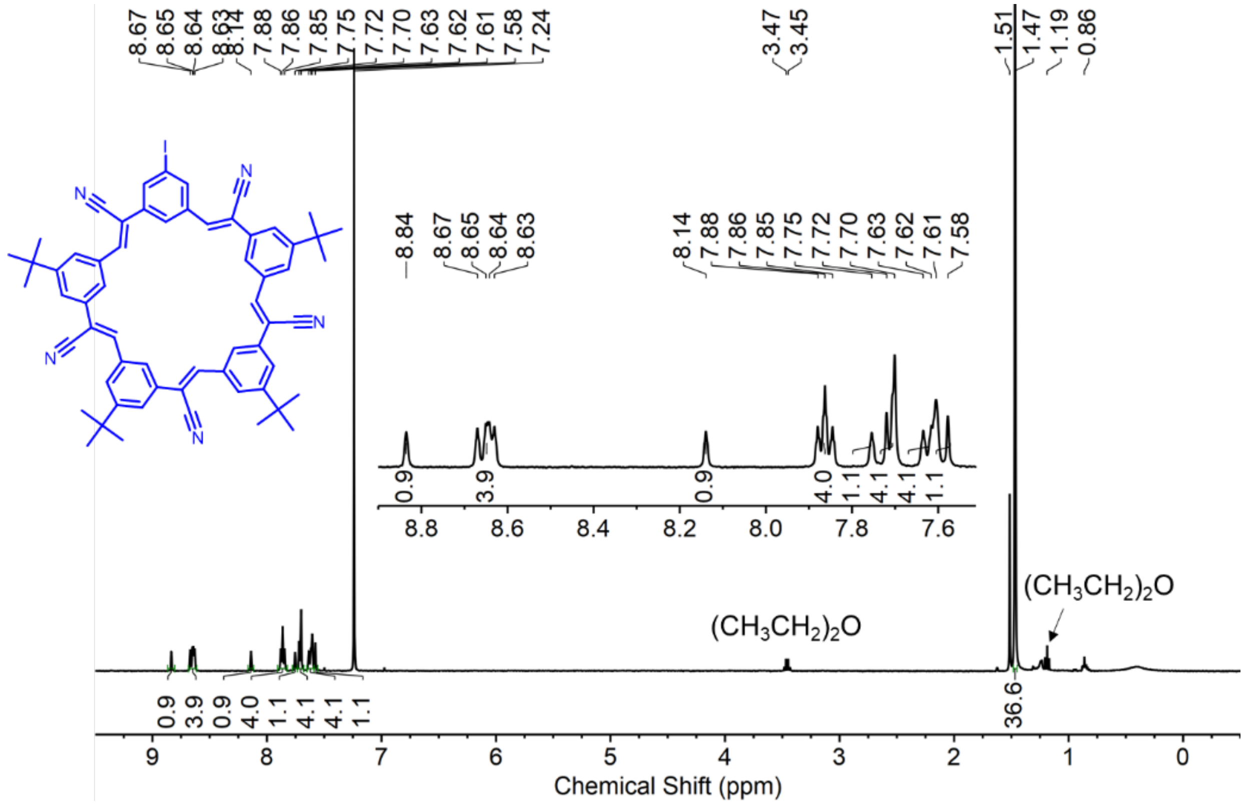

Figure S1. ^1H NMR spectra (400 MHz, $CDCl_3$) of the iodo-cyanostar macrocycle **1**.

^1H NMR (400 MHz, $CDCl_3$) of tris-alkoxy-5-ethynylbenzene precursor **2**: δ = 6.67 (s, 2H), 3.92 (m, 6H), 2.97 (s, 1H), 1.78-1.70 (m, 6H), 1.43 (m, 6H), 1.24 (m, 84H), 0.88-0.84 (t, J = 6.32 Hz, 9H).

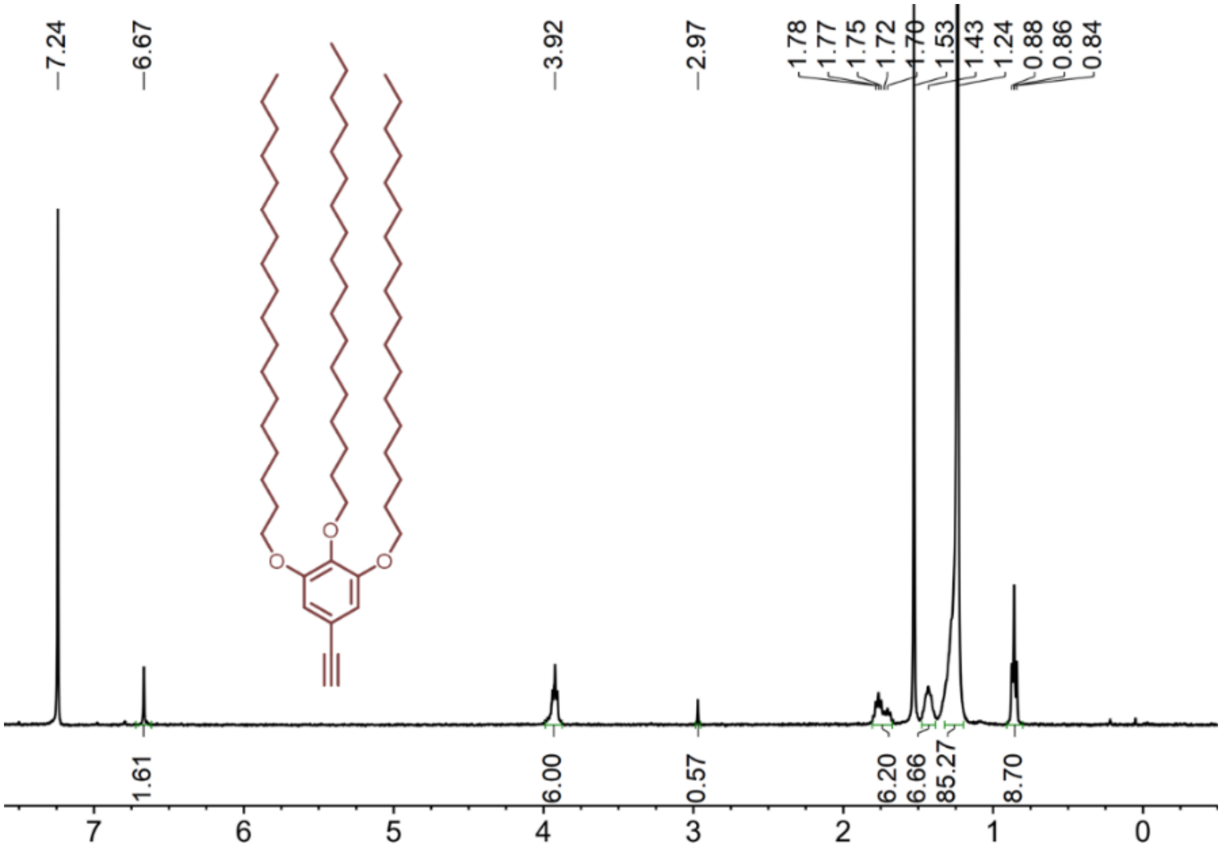

Figure S2. ^{1}H NMR spectra (400 MHz, $CDCl_3$) of tris-alkoxy-5-ethynylbenzene precursor **2**.

Cyanosurf 3: $Pd(PPh_3)_4$ (13 mg, 0.011 mmol), CuI (10.7 mg, 0.056 mmol) and iodo-cyanostar **1** (110 mg, 0.112 mmol) was dissolved in *iso*-propylamine ($i\text{-}Pr_2NH$, 5 mL) and THF (20 mL) under argon. A solution of tris-alkoxy-5-ethynylbenzene precursor **2** (101 mg, 0.112 mmol) in THF (100 mL) was added dropwise into the reaction mixture over one hour using an addition funnel at room temperature. The reaction mixture was stirred overnight and monitored by TLC until all the starting materials were consumed. The solvents were removed by reduced pressure and re-dissolved in dichloromethane (60 mL). The organic solution was washed with 1 M HCl (30 mL × 2), saturated NH_4Cl aqueous solution (30 mL × 2), and brine (30 mL × 2), respectively. The organic phase was separated, dried over Na_2SO_4 and filtered. The solvent was removed by reduced pressure and the residue was purified by column chromatography on SiO_2 (CH_2Cl_2: hexane, 2:1) to produce cyanosurf **3** as pale-yellow solid. (88 mg, 45%) ^{1}H NMR (500 MHz, $CDCl_3$): δ = 8.57 (s, 2H), 8.55 (s, 1H), 8.53 (s, 1H), 8.37 (s, 1H), 7.85 (d, J = 5.1 Hz, 2H), 7.82 (d, J = 6.5 Hz, 3H), 7.76 (s, 1H), 7.71 (s, 2H), 7.71 (s, 1H), 7.68 (s, 1H), 7.63 (m, 5H), 6.84 (s, 2H), 4.02 (t, J = 6.5 Hz, 6H), 1.83–1.80 (m, 4H), 1.78–1.72 (m, 2H), 1.53 (d, J = 3.5 Hz, 43H with H_2O peak), 1.28 (d, J = 4.8 Hz, 90H), 0.89 (t, J = 5.7 Hz, 9H) ppm. $^{13}C\{^{1}H\}$ NMR (126 MHz, $CDCl_3$): δ = 154.14, 154.08, 153.1, 142.5, 141.6, 141.5, 140.2, 139.6, 134.7, 134.3, 134.1, 133.8, 133.8, 133.7, 133.7, 133.4, 129.3, 127.7, 126.9, 126.7, 126.5, 126.2, 126.1, 126.0, 125.8, 124.6, 124.5, 117.4, 117.2, 117.1, 116.8, 112.1, 110.9, 110.8, 110.4, 109.6, 92.7, 86.6, 73.6, 69.3, 35.6, 32.0, 31.3, 30.0, 29.5, 29.4,

26.2, 22.7, 14.1 ppm. HR-FTMS (ESI) m/z: calcd. For $C_{123}H_{169}ClN_5O_3$ at $[M+Cl]^-$: 1799.2919; found: 1799.2870.

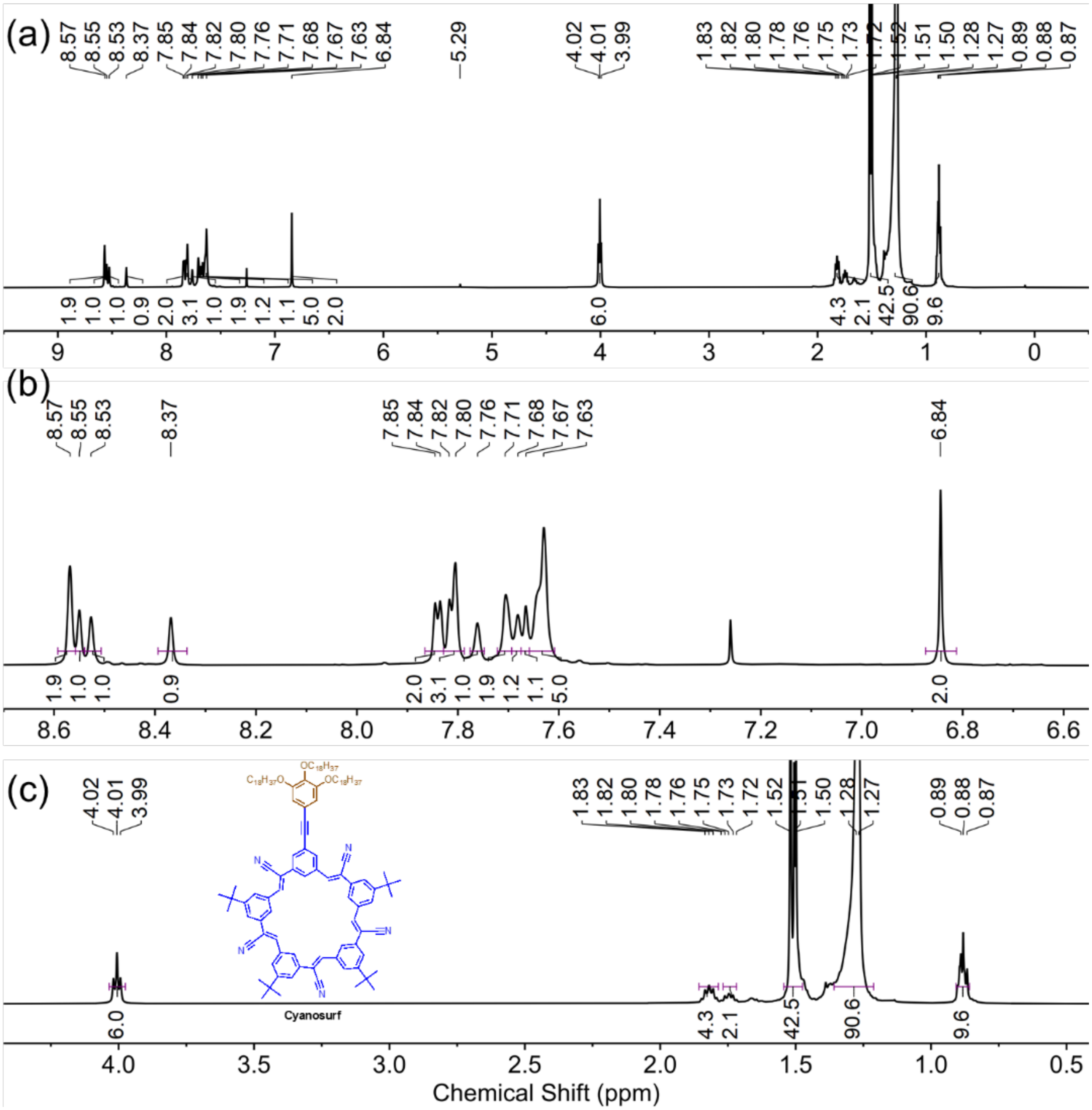

Figure S3. Stacked ^{1}H NMR spectra (400 MHz, $CDCl_3$) of the **cyanosurf** macrocycle with corresponding (a) full spectrum, (b) aromatic region and (c) aliphatic region.

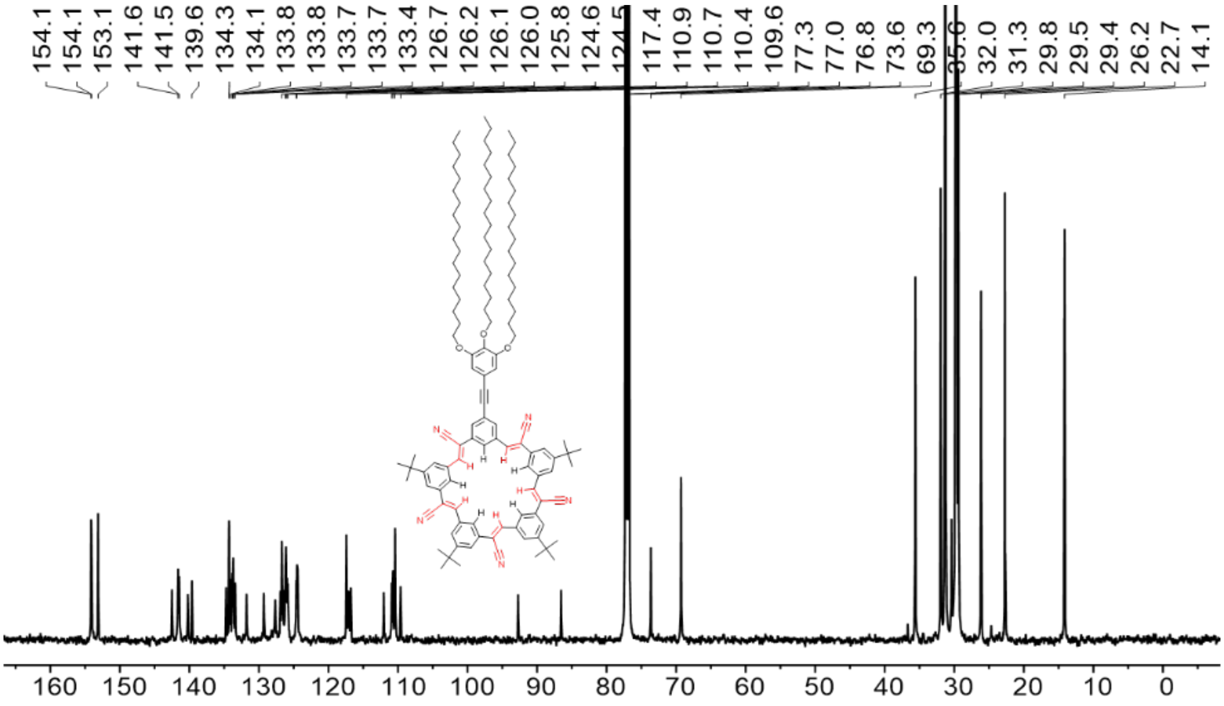

Figure S4. $^{13}C\{^{1}H\}$ NMR spectrum of the **cyanosurf** in $CDCl_3$. (125 MHz, 298 K)

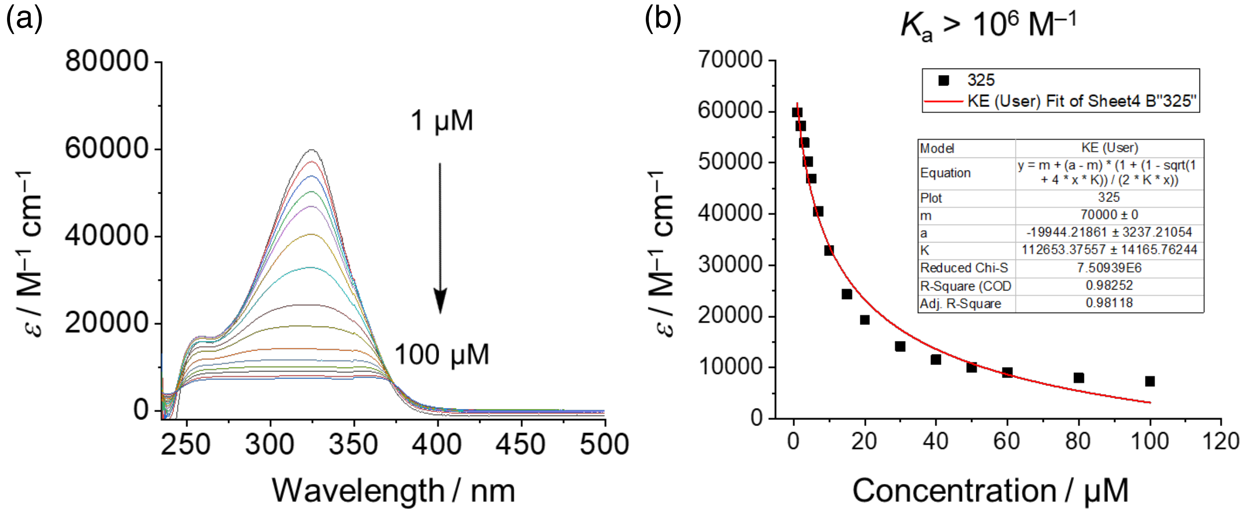

Figure S5. (a) Variable concentration UV-Vis extinction maxima of **cyanosurf** in $CHCl_3$. (b) The molar absorptivity value at 325 nm and its fitted curve based on the isodesmic equal-*K* model. The determined self-association constant (K_a) is over 10^6 M^{-1} indicative of large aggregation of the **cyanosurf** macrocycle in $CHCl_3$.

Based on the variable concentration UV-Vis study, we observed significant decrease of molar extinction coefficient along with increasing concentration from 1 μM to 100 μM (Figure S5a). By fitting the changes of extinction coefficient maxima at 325 nm, we can estimate an apparent self-association constant (K_a) based on the isodesmic indefinite self-association model

(*Chem. Rev.* **1996**, *96*, 3043). The obtained K_a is determined to be $(1.13 \pm 0.14) \times 10^5$ M^{-1}. However, as illustrated in Figure S5b, this model shows some deviation from the observation suggesting another model might be needed to obtain accurate self-association constants. Such models typically rely on nucleation and growth mechanisms of which the dimer model is the simplest (E. W. Meijer, *Chem. Rev.* **2009,** *109,* 5687–5754). However, distinguishing between such models requires evidence for the nucleation event, which is missing in the present case. For this reason, we have retained the use of the isodesmic model as a point of reference and comparison to related systems.

2. Experimental Methods for Surface Analysis

2.1 Materials

The sodium salts were commercially available and used without further purification. Sodium dihydrogen phosphate (monohydrated, 99.5%, Sigma), sodium chloride (99.5%, Sigma), sodium hexafluorophosphate (98.5%, Acros Organics) and sodium perchlorate (98%, Sigma) were dissolved in fresh ultrapure water with a resistivity of 18.2 MΩ cm (Milli-Q Advantage A10, EMD Millipore). Chloroform (HPLC grade, Fisher Scientific) was used to dissolve the **cyanosurf** macrocycle and deposit it onto the aqueous surface. The 10 mM phosphate, hexafluorophosphate, and perchlorate solutions had measured pH of 4.7, 4.01, and 6.99, respectively.

2.2 Instrumentation

All measurements are performed at ambient temperature 21.0 ± 2 °C, atmospheric pressure, and relative humidity 31 ± 7 %. Surface pressure-area (Π-A) isotherms were performed on a custom Teflon Langmuir trough with an area of 144.5 cm^2 equipped with movable Delrin barriers (KSV NIMA, Finland). Surface pressure was measured by the Wilhelmy plate method and controlled using KSV software (KSV NIMA, Finland). Prior to each experiment, the presence of extraneous surface-active contaminants was checked by sweeping the surface of the aqueous solution with the barriers until no significant change in the surface pressure (< 0.1 mN / m) was observed.

Surface Pressure-Area Isotherms.

Surface pressure- area (Π-A) isotherms of **cyanosurf** on neat water and salt solutions were performed on a computer-controlled Langmuir trough (KSV), this custom Teflon trough (168 mm x 85 mm) was equipped with two movable Delrin barriers (KSV NIMA, Finland) for symmetrical compression of the **cyanosurf**. Prior to each measurement, the trough needed to be cleaned with copious amounts of ethanol and Milli-Q water. The presence of organic contamination was checked by sweeping the surface of the subphase solution with a fast compression of the Delrin barriers at 227 mm per minute per barrier to ensure there was no significant rising of the surface pressure (<0.1 mN / m).

The **cyanosurf** solutions were deposited dropwise using the Hamilton syringe method and surface pressure was measured using Wilhelmy paper plates (Whatman, Ashless). After spreading, a 10-minute delay was applied to ensure solvent evaporation. Delrin barriers compressed symmetrically at a speed of 5 mm/min/barrier until the collapse of the monolayer was reached. For

IRRAS experiments, once the desired surface pressure was reached, the barriers are oscillated slowly at a rate of 1 mm / min / barrier both forward and backward to maintain the surface pressure.

Infrared Reflection−Absorption Spectroscopy

To probe the surface signature and organization of **cyanosurf** on the surface of subphase solutions, infrared reflection−absorption spectroscopy (IRRAS) spectra were collected using a Fourier transform infrared spectrometer (Perkin Elmer). The FT-IR spectrometer is equipped with a liquid nitrogen-cooled MCT (HgCdTe) detector and modified with a breadboard setup using two gold mirrors fixed at an incidence angle of 48° to collect the reflectivity off the **cyanosurf**. The IRRAS spectra were plotted as reflectance−absorbance (RA), which is given as RA = −log (R_c/R_0), where R_c is the reflectivity of the **cyanosurf** surface and R_0 is the reflectivity of the subphase solution (i.e., either water, ClO_4^-, or PF_6^-). An average of 300 scans over full range (450-4000 cm^{-1}) was collected using unpolarized light in the single-beam mode for each spectrum. Π-A isotherms and IRRAS spectra shown here are the result of averaging at least three spectra using the average function in Origin software (Origin 9, Northampton, MA).

Brewster Angle Microscopy

A custom-built Brewster angle microscope setup was used to simultaneously record Brewster angle microscopy (BAM) images and Π−A isotherms. The He-Ne laser source (1.5 mW, Research Electro-Optics, Boulder, CO) emitted polarized light at 543 nm with linear polarization. The light was filtered by a Glan polarizer for p-polarized purification before reaching to the aqueous surface. The incoming angle was first fixed at ∼53° for the aqueous solution surface. The reflected beam was collected after going through a 10× infinity-corrected super-long working distance objective lens (CFI60 TU Plan EPI, Nikon Instruments, Melville, NY) and collimated by a tube lens (MXA22018, Nikon Instruments; focal length 200 mm) before going into a back-illuminated EM-CCD camera (iXon DV887-BV, Andor Technology USA, Concord, MA; 512 × 512 active pixels with 16 μm × 16 μm pixel size). The BAM images were processed using ImageJ software77 and cropped from their original size to show the region of highest resolution. The dark regions of the images corresponded to the aqueous surface of subphase, whereas the light blue and bright regions of the images corresponded to the **cyanosurf** domains with low and high (aggregates) coverage, respectively.

Sum Frequency Generation Spectroscopy

The detail of the broadband SFG spectrometer set up used for this study was previously reported.[S3,S4] Briefly, a regenerative Ti:sapphire amplifier (Spitfire Ace, Spectra-Physics) seeded with a sub-50 fs 800 nm pulse from a Ti:sapphire oscillator provides an ∼4 W beam of 75 fs pulses and 1 kHz repetition rate. The amplified beam is then directed through a 50:50 beam splitter. One half of the beam is needed to pump an optical parametric amplification system (TOPAS-C, Light Conversion), which is coupled to a non-collinear difference frequency generator (NDFG, Light Conversion) to generate the broadband infrared beam. The other half of the beam intensity is spectrally narrowed to a fwhm of 12 cm^{-1} by an etalon (SLS Optics, United Kingdom) and is used as the visible 797 nm beam. The IR and the visible beams are co-propagating and fall on the sample

surface at an angle from the surface normal of 60 and 50 degrees, respectively. The IR beam is focused on the sample surface with a CaF_2 lens (15 cm FL) and the visible beam is focused approximately 1 cm after the surface with a BK7 lens (25 cm FL). The sum frequency signal is collected in the reflected direction by a spectrometer (IsoPlane SCT 320, Princeton Instruments) and an LN2 CCD (PyLoN, 1340 × 400 pixels, Princeton Instruments). All the spectral measurements are done in SSP polarization combination (for SF, vis, IR, respectively). The data for the OH spectral region was obtained with a 60 sec integration time and 5 averages. The spectra were then normalized to the non-resonant spectrum of a gold mirror (Thor labs) after background subtraction.

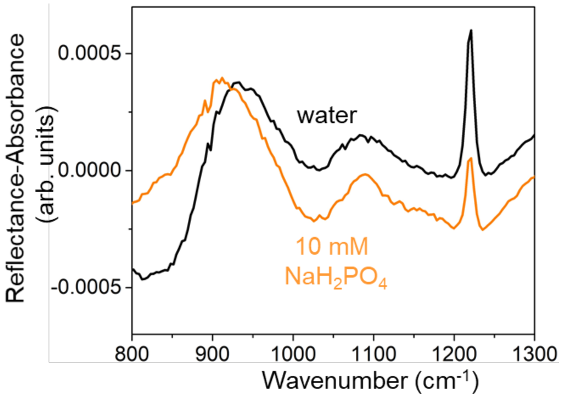

Figure S6. *The* IRRAS spectrum of surface **cyanosurf** molecules on water is shown as a black trace. The spectrum of surface **cyanosurf** molecules with 10 mM of the sodium phosphate at an MMA of 150 Å^2 / molecule is shown as an orange trace.

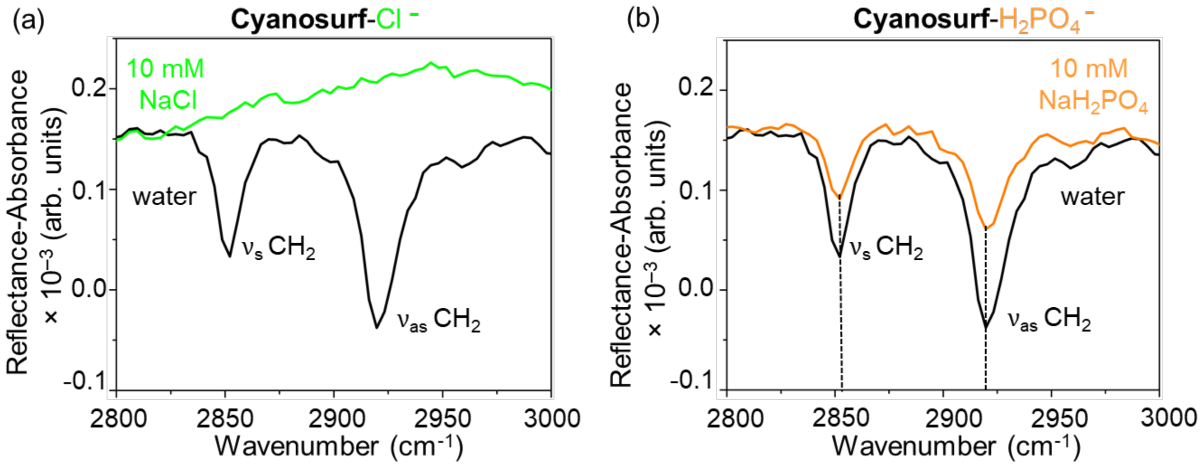

Figure S7. IRRAS spectra of surface **cyanosurf** molecules on water is shown as a black trace for (a, b). The spectra of the surface **cyanosurf** molecules with 10 mM of the sodium salts of (a) Cl^- (green) and (b) $H_2PO_4^-$ (orange) at a MMA of 150 Å^2 / molecule

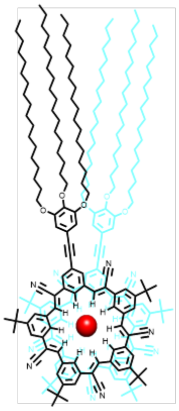

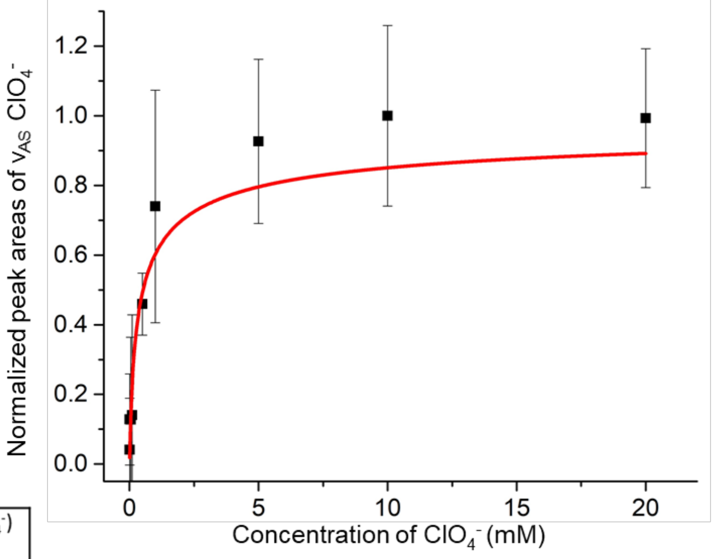

ClO_4^- Concentration (mM)	Normalized $v_{AS}(ClO_4^-)$ Peak Area
0.005	0.04 ± 0.15
0.01	0.13 ± 0.13
0.05	0.13 ± 0.24
0.1	0.14 ± 0.29
0.5	0.46 ± 0.09
1	0.74 ± 0.33
5	0.93 ± 0.24
10	1.00 ± 0.26
20	0.99 ± 0.20

Fit	Langmuir
Equation	$A = A_{max} \frac{1 + 8K_{a1}[S][Zn^{2+}] - \sqrt{1 + 16K_{a1}[S][Zn^{2+}]}}{8K_{a1}[S][Zn^{2+}]}$
R^2	0.95
A_{max}	0.99
Binding Affinity K (M^{-1})	$0.96 \pm 0.21 \times 10^3$

Figure S8. Langmuir titration curve showing normalized IRRAS peak intensity for the ClO_4^- asymmetric vibration (1110 cm^{-1}) obtained from the spectra of **cyanosurf** molecules on solutions with ClO_4^- at different concentrations. IRRAS data collected at a MMA of 150 $Å^2$ / molecule. Least-squares regression fitting was based on the equation associated with formation of a 2:1 **cyanosurf**-anion complex. Large uncertainties in the IRRAS intensity data are due to the inherent variability in the IRRAS spectral response and signal-to-noise factors. Reported error ($\pm 0.21 \times 10^3$ M^{-1}) corresponds to one standard deviation from the spectral fits.

References

S1. C. R. Benson, C. Maffeo, E. M. Fatila, Y. Liu, E. G. Sheetz, A. Aksimentiev, A. Singharoy and Amar H. Flood, *Proc. Natl. Acad. Sci. USA* 2018, **38**, 9391-9396.

S2. C. Zhu, T. Wang, C.-J. Su, S.-L. Lee, A. Rives, C. Duhayon, B. Kauffmann, V. Maraval, C. Chen, H.-F. Hsu and R. Chauvin, *Chem. Commun*. 2017, **53**, 5902-5905.

S3. E. M. Adams, D. Verreault, T. Jayarathne, R. E. Cochran, E. A. Stoneb and H. C. Allen, *Phys. Chem. Chem. Phys.,* 2016, **18**, 32345-32357.

S4. E. M. Adams, B, A. Wellen, R, Thiraux, S, K. Reddy, S. Vidalis, F. Paesanib and H. C. Allen, *Phys. Chem. Chem. Phys.*, 2017, **19**, 10481-10490.
